# Value-Added Products from Agro-Food Residues

**DOI:** 10.3390/foods11050766

**Published:** 2022-03-07

**Authors:** Ana Belen Diaz, Ana Blandino

**Affiliations:** Department of Chemical Engineering and Food Technology, Faculty of Sciences, IVAGRO, University of Cádiz, Campus Universitario de Puerto Real, 11510 Puerto Real, Spain; ana.blandino@uca.es

The agri-food sector produces large amounts of waste annually, most of which is lignocellulosic biomass. The growing concern about environmental issues, together with the diminishing of fossil resources, has driven the use of renewable and clean resources for the production of high-added-value products [1]. The use of lignocellulosic biomass for this purpose is promising, given that it represents the most abundant (200 billion tons/year) and renewable material in the world. Moreover, it does not compete with food production or animal feed [2]. Traditionally, its use has been limited to being burned for cooking and heating, causing serious environmental impacts such as land degradation and desertification [3,4].

The composition of lignocellulosic biomass is mainly comprised of carbohydrates, generally cellulose 40–60% and hemicellulose 10–40%, and lignin 15–30%, which are associated in a hetero-matrix and whose relative composition depends on the species, type or source of biomass [1,5]. Among these polymers, cellulose represents the core of the complex matrix, which is packed into bundled semi-crystalline microfibers [6]. Given that lignocellulosic biomass is compositionally rich in carbohydrates, it is a suitable resource for the production of bio-based products and chemicals [7].

This Special Issue comprises 10 selected papers in a wide range of related topics, including the pretreatment of lignocellulosic biomass, anaerobic digestion and the production of fermentable sugars and bioactive compounds such as fatty acids, antioxidants, dietary fiber, prebiotics and compounds for food packaging. One of the papers is focused on the application of several pretreatments on exhausted sugar beet pulp, including a biological one, an oxidant pretreatment with alkaline hydrogen peroxide and a thermochemical pretreatment with acid, to solubilize polysaccharides and facilitate the access to cellulose in the subsequent enzymatic hydrolysis for the production of sugars [8]. These sugars were further fermented with *Lactobacillus plantarum* for the production of lactic acid, a versatile organic acid widely used in the food industry. Despite the high percentage of carbohydrates in the lignocellulosic biomass structure, its recalcitrant structure hinders the access of enzymes and microbes. Thus, the application of a previous pretreatment stage significantly improves the process, as it enables the solubilization of the hemicellulose and lignin fractions, improving cellulose accessibility [9,10].

For the conversion of lignocellulosic biomass into fermentable sugars, a hydrolysis stage is needed, which can be carried out chemically or enzymatically, the latter being more environmentally friendly [11]. In this regard, one of the papers is based on the performance of the hydrolysis of lignocellulosic biomass by the direct addition of the fermented solid obtained by fungal solid-state fermentation as a source of enzymes [12]. The influence of several parameters on reducing the production of sugars was studied, including temperature, the fermented-to-fresh solid ratio, the supplementation of fermented biomass with commercial enzymes and the use of high-solid, batch-fed hydrolysis. With the proposed strategy, the enzyme extraction and purification stages can be avoided.

In another paper included in the Special Issue, an extrusion pretreatment with alkali was applied to vegetal tomato plant waste to concentrate the carbohydrate fraction and improve the enzymatic digestibility [13]. During extrusion, the biomass is subjected to heating, mixing and shearing stresses, which cause chemical and physical changes and have potential to improve enzymatic hydrolysis and anaerobic digestion for sugars and methane production, respectively [14]. In another paper in this Special Issue, several additives generated from different activity sectors in agriculture and industry were selected to enhance and stabilize the start-up of the semi-continuous anaerobic digestion of food waste [15]. It is known that this process shows many technical and economic limitations, given that food waste contains easily degradable components, which produce acidification, with weak buffer capacity, resulting in process inhibition. In this respect, the use of co-substrates represents an interesting approach [16]. The results shown in this paper demonstrated that both agricultural and industrial waste were very efficient at improving the anaerobic digestion of food waste or improving the quality of the effluent from the anaerobic digestion [15].

In addition to carbohydrates, agro-food residues are also an important source of natural bioactive compounds with high biological significance and economically feasible production. So, in one paper included in this Special Issue, the level of fatty acids, carotenoids and tocopherols were determined in apple seeds of different geographical origins and from different varieties of Norwegian cultivars [17]. This biomass has turned out to be a good source of fatty acids, linoleic acid, oleic acid and palmitic acid being dominant. Given its composition, the oil of apple seeds seems to have promise as an edible oil.

The residue generated from the processing of *Tacinga inamoena* (cumbeba) fruit pulp is also a source of bioactive compounds, which has been used as a raw material in another paper included in this Special Issue for the extraction of flavonoids, anthocyanins and betalains [18]. Convective drying was applied to the residue, which has turned out to be even better than freeze-drying for preservation until use and prevents the interference of water during the extraction process, improving the efficiency of the extraction of bioactive compounds. So, this paper also aims to study the influence of temperature and the drying method used to select the best mathematical model to calculate the drying rate, the effective diffusivity and the activation energy.

Another paper of this Special Issue is focused on the use of artichoke crop residues as raw materials for the production of valuable compounds, specifically phenols and inulin [19]. Inulin is a natural storage polysaccharide in plants that is considered a prebiotic compound which is indigestible by humans but stimulates the growth and activity of specific microorganisms of the colon [20]. In the processing of artichoke, large amounts of waste biomass (80–85%) are generated which are unsuitable for human consumption [21,22]. However, given that this residue includes beneficial compounds, the authors propose a sequential microwave extraction process inspired by green extraction techniques and solvents so that the extracts can be directly used for humans and animals.

In addition to inulin, xylooligosaccharides (XOS), which are composed of sugar oligomers of xylose units, are other prebiotics that can be obtained from agro-food residues [23]. The use of lignocellulosic biomass as raw material for their production is promising, given that it contains a large amount of xylan. So, in another paper of this Special Issue, XOS were produced by liquid hot water (LHW) treatment of the tropical hardwood *Eucalyptus pellita*, evaluating the influence of reaction temperature, time and pH [24]. LHW has shown to be more efficient than conventional processes, with higher cost and longer hydrolysis time required for xylanase utilization [25].

Inulin, xylooligosaccharides, beta-glucans, etc., belong to the ‘dietary fiber’ group, which shows a carbohydrate complex structure that escapes digestion and absorption in the upper human gastrointestinal tract, promoting health benefits to consumers [26,27]. This Special Issue includes a review of the methods used for dietary fiber fractionation to isolate starch and protein and obtain the fiber as a resulting product [28]. Moreover, the review provides a deep characterization based on the fiber’s structural, physicochemical and rheological properties. Finally, the paper describes the potential of defatted oilseeds (seeds whose fat contents have been removed partially or fully to subsequently improve the protein fraction) as a source of dietary fiber.

Recently, lignocellulose has also shown great potential for chemical cellulose modification and the use of its derivatives, such as nanocellulose, as additives and viscosity modifiers in food, pharmaceuticals, cosmetics and the development of thermoplastic polymers, among others [29]. In this regard, one of the papers of this Special Issue aims to develop films for food packaging with polyvinyl alcohol (synthetic biopolymer) reinforced with lignocellulose nanofibers obtained by the mechanical and chemical pre-treatment of woody residues from tomato, eggplant and pepper plants. The reinforced films showed better properties, including thermal resistance, mechanical parameters, water vapor permeability, UV blocking, antioxidant activity, etc.
